# Adjustable mini-sling compared with conventional mid-urethral slings in women with urinary incontinence: a 3-year follow-up of a randomized controlled trial

**DOI:** 10.1007/s00192-019-04004-w

**Published:** 2019-06-20

**Authors:** Vasileios Alexandridis, Martin Rudnicki, Ulf Jakobsson, Pia Teleman

**Affiliations:** 1 Department of Obstetrics and Gynecology, Skane University Hospital, Lund, Sweden; 2 Faculty of Medicine, Lund University, Lund, Sweden; 3 Department of Obstetrics and Gynecology, Skane University Hospital, Jan Waldenströms gata 47, 214 66 Malmö, Sweden; 4 Department of Obstetrics and Gynecology, Odense University Hospital, Odense, Denmark; 5 Department of Clinical Research, University of Southern Denmark, Odense, Denmark; 6 Center for Primary Healthcare Research, Clinical Research Center, Malmö, Sweden

**Keywords:** Mid-urethral sling, Mini-sling, Single-incision sling, Stress urinary incontinence

## Abstract

**Introduction and hypothesis:**

The aim of this study was to compare the long-term subjective outcomes of an adjustable single-incision sling (Ajust®) vs standard mid-urethral slings (SMUS) for the treatment of women with stress urinary incontinence.

**Methods:**

This study was designed as a multicenter prospective randomized trial. Women under 60 years old with objectively verified stress urinary incontinence were included from seven centers in three countries. Women with mixed urinary incontinence were also included. Randomization was held in blocks for operation with either Ajust® or SMUS. Women analyzed at 1-year follow-up received the International Consultation on Incontinence Questionnaire Urinary Incontinence Short Form (ICIQ-UI-SF), International Consultation on Incontinence Questionnaire Overactive Bladder, Pelvic Organ Prolapse/Urinary Incontinence/Sexual Function Questionnaire-12, Patient Global Impression of Severity, and Patient Global Impression of Improvement questionnaires, together with a bladder diary to fill out at least 3 years after the procedure. The main outcome evaluated was the subjective cure rate as reported through the ICIQ-UI-SF questionnaire at 3 years.

**Results:**

In total, 205 women participated in the 3-year follow-up: 107 in the Ajust® and 98 in the SMUS group. No significant difference was observed between the groups regarding subjective cure rate (50.9% vs 51.5%, *p* = 0.909) or dyspareunia. Both groups demonstrated similar postoperative perception of improvement in addition to reduced urgency and urge urinary incontinence. The postoperative improvement remained at the same level after 3 years as it was at 1-year follow-up for both Ajust® and SMUS.

**Conclusions:**

Ajust® appears to be equally effective and safe as SMUS with regard to long-term follow-up of patient-reported outcomes.

## Introduction

Single-incision mid-urethral slings (SIMS), or mini-slings, have been introduced over the past few years to minimize the surgical procedure and operation time and decrease the number of complications associated with surgery for the treatment of stress urinary incontinence (SUI). A recent meta-analysis reported insufficient results for the extraction of safe conclusions, but there is some evidence indicating better performance of the SIMS in terms of post-operative pain and duration of the operation [1]. On the other hand, the TVT-Secur has consistently demonstrated inferior results compared with standard mid-urethral slings (SMUS) and has been withdrawn from the market [2, 3]. These findings indicate the importance of the fixation system associated with each sling, as this aspect is suggested to be the main reason for the failure of the TVT-Secur [1]. Furthermore, long-term follow-up research is needed before introducing a new device into clinical practice. Such data concerning the long-term efficacy and safety of SIMS has, to our knowledge, not yet been published.

The lack of data is also apparent concerning the new device Ajust®, which is an adjustable mini-sling that works in a similar manner to SMUS, as the tension of the sling can be modified during the procedure. Several studies have evaluated the short- to midterm efficacy of Ajust®, demonstrating a subjective cure rate of 77–94% and a complications profile similar to that of trans-obturator vaginal tape (TVT-O) [4–17]. There is some evidence indicating greater cost-effectiveness compared with TVT-O [18] and Ajust® has been shown to be easier to attach to the obturator complex than other mini-slings [19]. However, most of the studies concerning Ajust® demonstrate a follow-up period of around 1–2 years, with the exception of one study analyzing 3-year results [20]. Considering the initial promising results of the Ajust® sling, Rudnicki et al. published a randomized controlled trial in 2017, which was the first to compare Ajust® with SMUS other than TVT-O [21]. This study showed no significant difference in objective or subjective cure rates after 1 year and there was no difference observed in postoperative improvement or complication rates either. As early as during the design of this study, a 3-year follow-up was planned.

The primary aim of the present study was to compare the long-term subjective cure rate of Ajust® versus SMUS. The secondary aim was to compare the patient-reported improvement, complications, and symptoms regarding urinary incontinence and pelvic floor function 3 years after undergoing surgery with Ajust® versus SMUS.

## Materials and methods

This study was designed as a prospective multicenter randomized clinical trial. It was funded by the Nordic Federation of Obstetrics and Gynecology (NFOG) Research Fund after an external evaluation by the NFOG Scientific Committee and approval by the NFOG Board. The funder was not involved in conducting this study in any way. Ajust® (Bard, Murray Hill, NJ, USA) was compared with the conventional mid-urethral slings tension-free vaginal tape (TVT) (Ethicon, Somerville, NJ, USA), TVT-O inside-out (Ethicon), and trans-obturator tape (TOT; Monarc; AMS, Minneapolis, MN, USA), depending on the type of the sling that each of the eight centers in Sweden, Denmark, and Norway preferred to use.

Randomization was carried out through a computer-generated list in blocks of 25 corresponding to each center in a ratio of 1:1 to either Ajust® or SMUS. A random allocation sequence was generated by an independent statistician and sealed, non-transparent envelopes were used during randomization. Data were recorded following inclusion. The follow-up conducted from inclusion up to 1 year is described in the study by Rudnicki et al. [21].

All women received oral and written information about the study protocol in advance and gave their consent to participation. Women participating had a medical history of either SUI, defined as the complaint of involuntary leakage of urine due to physical exertion, or mixed urinary incontinence (MUI), corresponding to women exhibiting SUI along with leakage associated with urgency. SUI had to be the dominating symptom and it was confirmed in all women included in the study by a positive standardized cough test with 300 cm^3^ water in the bladder. Medical history and physical examination were part of the pre-operative assessment and pelvic floor muscle training had failed or women had declined it.

Women were excluded if they were aged >60 years, had predominantly urge urinary incontinence (UUI), pelvic organ prolapse (Pelvic Organ Prolapse Quantification stage ≥2), previous incontinence or pelvic organ prolapse surgery, planned or present pregnancy, residual urine volume > 100 ml, previous pelvic irradiation, repeated urinary tract infections (four or more during the previous year), neurological conditions such as multiple sclerosis, current treatment with corticoids, inability to understand the protocol, and a history of genital or abdominal cancer or a pelvic mass.

All centers except for one participated in the 3-year follow-up, i.e., seven centers. One center did not participate because of local logistics issues. All women who participated in the 1-year follow-up received questionnaires at least 3 years after the Ajust® or SMUS procedure, which they were asked to fill out and submit. Along with the questionnaires, they received a bladder diary for 2 consecutive days. Women who did not respond were contacted with letters before exclusion.

All surgeons were specialized gynecologists and all had performed more than 100 SMUS procedures and at least two operations with Ajust® under an instructor’s supervision before entering the study. The women received local, spinal or general anesthesia, depending on local regimens, and cystoscopy was performed peri-operatively in all cases. The clinical part of the study, including selection of women and interventions, was carried out between May 2012 and April 2014.

No core outcome set regarding female pelvic floor disorders was available during the design phase of this study, but the development of one is currently ongoing. The outcomes chosen were in agreement with the International Consultation on Incontinence and the International Urogynecological Association recommendations for the evaluation of urinary incontinence and pelvic floor function in women [22, 23]. The International Consultation on Incontinence Questionnaires (ICIQ), Urinary Incontinence–Short Form (ICIQ-UI-SF) and Overactive Bladder (ICIQ-OAB), the Patient Global Impression of Improvement (PGI-I) and the Patient Global Impression of Severity (PGI-S) questionnaires and, if sexually active, the Pelvic Organ Prolapse/Urinary Incontinence/Sexual Function Questionnaire (PISQ-12) were used together with a 2-day bladder diary for the evaluation of the effect of the procedures on the women’s pelvic tract and bladder symptoms in addition to quality of life parameters.

### Statistical analysis

The study was designed as a randomized trial and one-sided power analysis that was conducted before randomization showed that the inclusion of 131 women was required in each arm anticipating a cure rate of not less than 9% for Ajust® compared with SMUS (α = 0.05, β = 0.2) [21]. Data were analyzed using descriptive and analytical statistics. When analyzing descriptive data, mean, standard deviation, and range were used for continuous data, whereas median, interquartile range, and frequencies (*n* and percentage) were used for ordinal and nominal data. Student’s *t* test was used for normally distributed data and, for ordinal and non-normally distributed data, the Mann–Whitney *U* test or the Friedman test was used for comparison between groups. Post-hoc analysis of the Friedman test results was conducted using the Wilcoxon signed-rank test with a correction applied using the Bonferroni method (α = 0.017). The Chi-squared test and Fisher’s exact test were used for nominal data. All analyses were performed using IBM SPSS statistics (version 24) and the statistical significance was set at <0.05 (except for the post-hoc tests).

### Ethics approval

Ethics approval of the study was obtained from the local ethics committee in each country; Denmark ref. no. SJ252 (approved by the local ethics committee of Region Zealand, Denmark), Sweden ref. no. 2011/529, 2012/42 (approved by the regional ethics committee in Lund, Sweden), Norway ref. no. 2011/2005A (Ethics committee of Norway, REK).

The trial was registered at ClinicalTrials.gov: NCT01754558.

URL: clinicaltrials.gov/ct2/show/NCT01754558?term=NCT01754558&rank=1

## Results

**Fig. 1:**
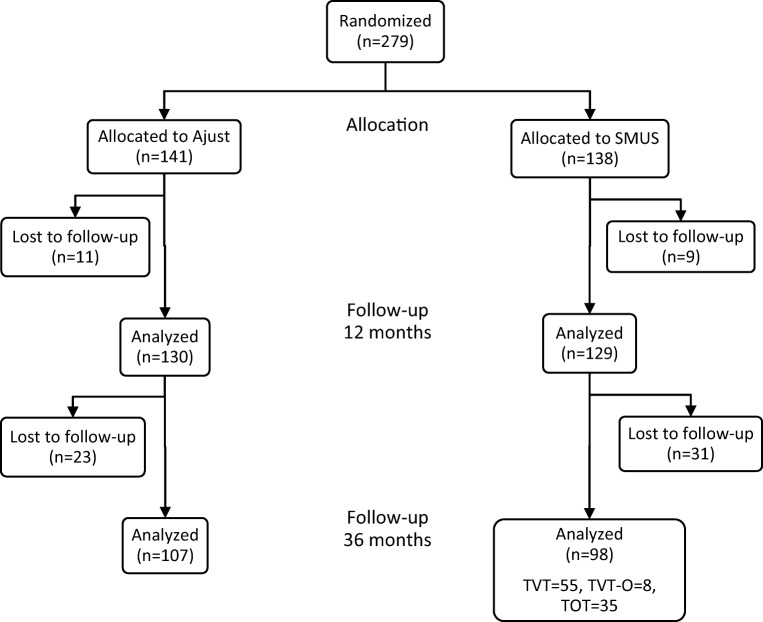
Flow diagram of the study population. *SMUS* standard mid-urethral sling, *TVT* tension-free vaginal tape, *TVT-O* trans-obturator vaginal tape, *TOT* trans-obturator tape

In total, 279 women from the seven centers in Sweden, Denmark, and Norway that participated in the 3-year follow-up were initially randomized between Ajust® and SMUS. Of those women, 259 were enlisted for 1-year follow-up. The results of 205 women were further analyzed 3 years after the Ajust® or SMUS operation, corresponding to 73% of the women analyzed in the 1-year follow-up study [21]. The Ajust® group consisted of 107 women and the SMUS group of 98, which was equivalent to 76% and 71% respectively of those initially included. Detailed information, in addition to the distribution among the various SMUS, is shown in Fig. 1. The women in both groups had the same characteristics at baseline and similar bladder function (Table 1).

**Table 1:** Descriptive characteristics at baseline of the 279 participants

	Ajust (*n* = 141)	SMUS (*n* = 138)	*p* value
Age (years), mean ± SD (range)	44.9 ± 6.8 (27–59)	45.9 ± 7.3 (27–60)	0.228*
BMI (kg/m^2^), mean ± SD (range)	26.2 ± 4.8 (19–49)	26.6 ± 4.6 (16–42)	0.550*
Parity, median (IQR)	2.0 (1.0)	2.0 (1.0)	0.697**
Postmenopausal, *n* (%)	28 (20.3)	32 (24.4)	0.415**
On HRT, *n* (%)	15 (10.6)	18 (13.1)	0.519**
– Systemic HRT, *n* (%)	6 (17.6)	5 (14.7)	0.745**
– Vaginal HRT, *n* (%)	10 (30.3)	13 (37.1)	0.551**
Smokers, *n* (%)	25 (18.0)	21 (15.4)	0.572**
Diabetes, *n* (%)	0 (0.0)	4 (2.9)	0.060***
Chronic obstructive lung disease, *n* (%)	3 (2.2)	2 (1.5)	0.503***
Previous hysterectomy, *n* (%)	15 (10.7)	15 (10.9)	0.967**
Previous prolapse surgery, *n* (%)	3 (2.1)	2 (1.4)	0.507***
– Anterior	2 (66.9)	2 (100)	
– Anterior and posterior	1 (33.3)	0 (0.0)	
Medication, *n* (%)			
– Diuretics	3 (2.1)	4 (2.9)	0.722***
– Antidepressants	8 (5.8)	6 (4.4)	0.603**
Stress urinary incontinence, *n* (%)	104 (74.8)	108 (80.6)	0.252**
Mixed urinary incontinence, *n* (%)	36 (26.1)	29 (21.2)	0.337**
Number of micturitions per day, mean ± SD (range)^a^	7.2 ± 2.1 (3–15)	7.0 ± 1.7 (3–13)	0.259*
Incontinence episodes per day, mean ± SD (range)^a^	3.2 ± 3.4 (0–24)	3.3 ± 4.0 (0–26)	0.721*
ICIQ score, median (IQR)	15.0 (5.0)	15.0 (4.0)	0.106****
Residual volume, median (IQR)	5.0 (30.0)	2.5 (19.3)	**0.037** ****
Flow max rate (ml/s), mean ± SD	27.9 ± 8.5	27.6 ± 12.5	0.897*
Dyspareunia, PISQ-12 (%)			0.505****
– Always	1.6	0.9	
– Usually	2.5	2.6	
– Sometimes	21.3	23.1	
– Seldom	30.3	35.0	
– Never	44.3	38.5	

Text in bold indicates statistical significance

*SMUS* standard mid-urethral sling, *BMI* body mass index, *IQR* interquartile range, *HRT* hormone replacement therapy, *ICIQ* International Consultation on Incontinence Questionnaire, *PISQ-12* Pelvic Organ Prolapse/Urinary Incontinence/Sexual Function Questionnaire

*Student’s *t* test

**Chi-squared test

***Fisher’s exact test

****Mann–Whitney *U* test

^a^Calculated from the bladder diary

The 3-year follow-up showed no difference in the subjective cure rate, which was defined as the proportion of women reporting no leakage at all in the ICIQ-UI-SF. The percentage of women who stated having no urinary incontinence whatsoever 3 years after the procedure was 50.9% vs 51.5% for the Ajust® vs the SMUS group respectively (*p* = 0.909). The corresponding cure rates based on the 2-day bladder diary were 71.1% for the Ajust® versus 82.9% for the SMUS group with no significant difference either (*p* = 0.481). However, women in the Ajust® group demonstrated a significantly lower number of micturitions per day (6.1 vs 6.7; *p* = 0.040; Table 2).

**Table 2:** Results at 36-months follow-up

	Ajust (*n* = 107)	SMUS (*n* = 98)	*p* value
Subjective cure rate, ICIQ-UI-SF (%)			
Question 3: “How often do you leak urine?”			0.909*
– Never	50.9	51.5	
– About once a week or less often	31.1	32.0	
– Two or three times a week	12.3	10.3	
– Once daily	3.8	1.0	
– Several times a day	1.9	5.2	
– All the time	–	–	
ICIQ score sum, questions 3–5 (mean ± SD)	2.8 ± 3.6	3.0 ± 3.9	0.660**
Number of micturitions per day (mean ± SD)	6.1 ± 1.4	6.7 ± 1.8	**0.040** **
Incontinence episodes, bladder diary, *n* (%)			0.481*
– Zero episodes	27 (71.1)	29 (82.9)	
– One episode	5 (13.2)	3 (8.6)	
– Two episodes	4 (10.5)	1 (2.9)	
– Three episodes	1 (2.6)	–	
– Four episodes	–	2 (5.8)	
– > Four episodes	1 (2.6)	–	
Dyspareunia, PISQ-12 (%)			0.496*
– Always	0.0	1.3	
– Usually	4.5	1.3	
– Sometimes	15.7	10.0	
– Seldom	22.5	41.3	
– Never	57.3	46.3	
PISQ-12 score sum (mean ± SD)	36.1 ± 3.7	35.1 ± 3.7	0.086*

Text in bold indicates statistical significance

*Mann–Whitney *U* test

**Student’s *t* test

The ICIQ-UI-SF score sum was equally reduced in both groups at 3-year follow-up (*p* = 0.660; Table 2) and equal over-the-time improvement was also observed regarding urgency and UUI based on the ICIQ-OAB questions 5a and 6a (Table 3). Both groups demonstrated the same degree of improvement as stated in the PGI-I questionnaire, with the percentage of women reporting significantly or much improved 3 years after the operation with Ajust® being 95.1% versus 89.7% for the women who received an SMUS. For both groups the improvement increased through the first post-operative year, remaining stable at the same level during the 3-year follow-up. The same pattern was observed using the PGI-S questionnaire with equal degrees of improvement of the severity status after 3 years between the groups, which was stable compared with the 1-year results (Table 4). Similarly, there was no difference observed in the percentage of women reporting dyspareunia after the operation or in the PISQ-12 score sum (Table 2). No major adverse events other than those reported at the 1-year follow-up were later recorded.

**Table 3:** International Consultation on Incontinence Questionnaire Overactive Bladder (ICIQ-OAB) results of the 205 women analyzed after 36 months

	Baseline	12 months	36 months	*p* value (post-hoc)
ICIQ-OAB, Question 5a (Ajust) %				**< 0.001** *
“Do you have to rush to the toilet to urinate?”				**(A, B)**
– Never	15.8	31.1	28.3	
– Rarely	33.7	43.7	48.1	
– Sometimes	38.6	19.4	17.9	
– Often	10.9	4.9	4.7	
– Always	1.0	1.0	0.9	
ICIQ-OAB, Question 5a (SMUS) %				**< 0.001** *
“Do you have to rush to the toilet to urinate?”				**(A, B)**
– Never	14.0	29.9	28.6	
– Rarely	36.6	46.0	37.8	
– Sometimes	44.1	24.1	28.6	
– Often	5.4	–	5.1	
– Always	–	–	–	
ICIQ-OAB, Question 6a (Ajust) %				**< 0.001** *
“Does urine leak before you can get to the toilet?”				**(A, B)**
– Never	21.6	50.4	48.1	
– Rarely	27.0	35.0	31.1	
– Sometimes	36.0	12.2	18.9	
– Often	12.6	2.4	1.9	
– Always	2.7	–	–	
ICIQ-OAB, Question 6a (SMUS) %				**< 0.001** *
“Does urine leak before you can get to the toilet?”				**(A, B)**
– Never	22.4	56.5	50.0	
– Rarely	36.4	33.0	30.2	
– Sometimes	33.6	7.8	15.6	
– Often	5.6	1.7	3.1	
– Always	1.9	0.9	1.0	

Post-hoc test: significant difference between A = baseline and 12 months, B = baseline and 36 months, C = 12 and 36 months. Text in bold indicates statistical significance

*Friedman test with Wilcoxon signed-rank test as post-hoc, Bonferroni method → α = 0.017

**Table 4:** Patient Global Impression of Severity (PGI-S) and Patient Global Impression of Improvement (PGI-I) results of the 205 women analyzed after 36 months

	Ajust baseline	Ajust 12 months	Ajust 36 months	SMUS baseline	SMUS 12 months	SMUS 36 months
PGI-S, %*						
– Normal	28	72.3	68.3	31.9	86.4	70.1
– Minor	5.0	25.7	27.9	12.1	13.6	22.7
– Moderate	40.0	2.0	2.9	38.5	–	6.2
– Severe	27.0	–	1.0	17.6	–	1.0
PGI-I, %**						
– Very much improved	–	75.0	71.6	–	89.5	75.3
– Much improved	–	19.0	23.5	–	4.7	14.4
– Minimally improved	–	4.0	3.9	–	3.5	7.2
– Unchanged	–	2.0	–	–	1.2	2.1
– Minimally worse	–	–	–	–	–	–
– Much worse	–	–	–	–	–	1.0
– Very much worse	–	–	1.0	–	1.2	–

Ajust: *n* = 107, SMUS: *n* = 98

*Comparison of PGI-S between Ajust and SMUS at baseline; ***p* = 0.033**, 12 months; *p* = 0.115 and 36 months; *p* = 0.913 (Mann–Whitney *U* test)

**Comparison of PGI-I between Ajust and SMUS at 12 months; ***p* = 0.028** and 36 months; *p* = 0.759 (Mann–Whitney *U* test)

## Discussion

### Main findings

Based on the results of this study, Ajust® demonstrates equal efficacy and safety to the widely used mid-urethral slings, whose characteristics have been confirmed by years of clinical experience and long-term follow-up trials. Furthermore, both methods seem to have a positive effect on overactive bladder (OAB) symptoms. This has been shown in other studies evaluating the effect of surgical treatment for MUI and it has been used as an argument by those supporting a common urethrogenic mechanism as the cause of both SUI and UUI [24]. Compared with the 1-year results from the study of Rudnicki et al. [21], the 3-year outcomes do not differ substantially regarding the cure rates, the OAB symptoms, the perception of improvement or the dyspareunia for either Ajust® or SMUS. This is confirmed by the analysis of the outcomes in our group of women over time, suggesting an invariable efficacy and safety of Ajust® after the first year and up to 3 years. The same was observed through long-term follow-up studies of conventional TVT slings that demonstrated only a small decrease in their effect over the years [25].

### Strengths and limitations

There are some limitations regarding this study. Our results are patient-reported and they are limited to 3 years after the operation. Objective data could not be obtained owing to a lack of alignment among the centers participating in the study. Sensitivity analysis was not done as we did not observe any tendency for any of the groups to consistently overcome the others and there were no signs of non-significant *p* values due to the sample size. Selection bias may occur, but the number of women missing at the 3-year follow-up is balanced between the two groups. In fact, the women who did not respond to our invitations were of lower age in both groups. One explanation may be that a large number of those women did not respond because they were satisfied with the operation and in that way we may have underestimated to some extent the cure rates in both groups. We have no reason to believe that the one missing center in particular would affect the outcomes of the study, based on the 1-year results. The women included in this study were under 60 years of age and the conclusions do not apply beyond this age group.

The strength of our study lies in the large number of women recruited, in addition to the multicenter, randomized design, which allows the comparison of Ajust® with other established mid-urethral slings. Additionally, in our study not just one sling was used in the control group, but each center used the type of sling they preferred, making it easier to generalize the conclusions. A recent meta-analysis has in fact demonstrated that all three SMUS used in our study are equally effective [26]. Moreover, Ajust® was not proven to be inferior to SMUS, despite the fact that surgeons were not equally familiar with the Ajust® procedure.

### Interpretation

This study is one of only two evaluating the performance of Ajust® separately and one of few evaluating the performance of adjustable mini-slings as a group for periods longer than 2 years [20, 25–27]. Therefore, the comparison of our results with those of other studies presents significant difficulties. Kluz et al. [20] reported recently in a retrospective design on the outcomes of Ajust® 36–50 months after the operation. The post-operative evaluation was carried out through a combination of interview and examination and the observed cure rate of 51.6% is in agreement with our results based on the ICIQ-UI-SF. However, the cure rates reported by the remaining studies evaluating the efficacy of Ajust® up to 2 years are significantly higher at around 85% [4–17]. On the other hand, the cure rate we observed of 71.1–82.9% based on the bladder diary was comparable with these studies and the same was observed for the percentage of women who reported being significantly/much better after both procedures, which was up to 95.1%. This discrepancy can be justified by the more objective character of the bladder diary in addition to its shorter period of registration, probably resulting in higher specificity, whereas the ICIQ provides a more comprehensive impression of the women’s symptoms, which in turn may generate greater sensitivity for detecting incontinence and, therefore, lower cure rates. After all, the correlation between data derived from bladder diaries and questionnaires regarding urinary incontinence is generally known to be weak [28]. However, it is to be noted that the bladder diary was submitted by significantly fewer women than those who answered the questionnaires.

In addition to the different ways in which cure is defined by each study group, variation in follow-up periods and study populations possibly also contributes to the different outcomes. The inclusion of women with MUI in our study certainly lowers the percentage of those reporting no leakage at all after the procedure, which was our primary outcome. In any case, the randomized design allows us to compare Ajust® with other slings with an established effect on incontinence, showing no significant difference, which is in agreement with the results of the other studies comparing Ajust® with TVT-O [7, 9–11, 13, 15–17]. Our findings are also in accordance with the results of meta-analyses regarding patient-reported cure rates for both short- and long-term follow-up periods, when comparing conventional mid-urethral slings with SIMS all together and with Ajust separately [29], likewise when comparing SMUS with the group of adjustable SIMS [30].

Ajust®, along with other mesh devices, has recently been withdrawn from the market. The manufacturing company declares only business reasons for this removal and no safety concerns whatsoever, asking at the same time for the immediate removal even of the unused devices. It remains to be seen if the results of long-term follow-up trials could have an impact on this decision. It is worth mentioning that this study was not funded in any way by the industry. We believe that such data should actually be provided by the company introducing a device and not by a study group interested in the security and efficacy of a new treatment available commercially. However, studies like this are important to minimize the risk of widely using a device without knowledge of potential adverse effects, in addition to acting as a springboard for future development.

## Conclusion

Our study shows that Ajust® is not inferior to the conventional mid-urethral slings regarding long-term follow-up periods. Our results, combined with the association of the failure of other mini-slings mainly with their inadequate fixation system, indicate that the presence of an efficient fixation in the obturator complex makes the potentially harmful penetration into deeper layers unnecessary. Further research is needed with longer follow-up periods and objective data to examine the possible benefits of mini-slings and gradually even the development of better devices.

## Abbreviations

ICIQ: International Consultation on Incontinence Questionnaire
ICIQ-OAB: International Consultation on Incontinence Questionnaire Overactive Bladder
ICIQ-UI-SF: International Consultation on Incontinence Questionnaire Urinary Incontinence Short Form
MUI: Mixed urinary incontinence
NFOG: Nordic Federation of Obstetrics and Gynecology
OAB: Overactive bladder
PGI-I: Patient Global Impression of Improvement
PGI-S: Patient Global Impression of Severity
PISQ-12: Pelvic Organ Prolapse/Urinary Incontinence/Sexual Function Questionnaire
SIMS: Single-incision mid-urethral slings
SMUS: Standard mid-urethral slings
SUI: Stress urinary incontinence
TOT: Trans-obturator tape
TVT: Tension-free vaginal tape
TVT-O: Trans-obturator vaginal tape
UUI: Urge urinary incontinence
